# Differential Expression of Myogenic and Calcium Signaling-Related Genes in Broilers Affected With White Striping

**DOI:** 10.3389/fphys.2021.712464

**Published:** 2021-07-26

**Authors:** Caroline Michele Marinho Marciano, Adriana Mércia Guaratini Ibelli, Jorge Augusto Petroli Marchesi, Jane de Oliveira Peixoto, Lana Teixeira Fernandes, Igor Ricardo Savoldi, Kamilla Bleil do Carmo, Mônica Corrêa Ledur

**Affiliations:** ^1^Programa de Pós-Graduação em Zootecnia, Universidade do Estado de Santa Catarina (UDESC-Oeste), Chapecó, Brazil; ^2^Embrapa Suínos e Aves, Concórdia, Brazil; ^3^Programa de Pós-Graduação em Ciências Veterinárias, Universidade Estadual do Centro-Oeste, Guarapuava, Brazil; ^4^Departamento de Genética, Universidade de São Paulo – Faculdade de Medicina de Ribeirão Preto, Ribeirão Preto, Brazil; ^5^Universidade do Contestado, Concórdia, Brazil

**Keywords:** gene expression, glycogen metabolism, pectoral myopathy, chickens, quantitative PCR

## Abstract

White Striping (WS) has been one of the main issues in poultry production in the last years since it affects meat quality. Studies have been conducted to understand WS and other myopathies in chickens, and some biological pathways have been associated to the prevalence of these conditions, such as extracellular calcium level, oxidative stress, localized hypoxia, possible fiber-type switching, and cellular repairing. Therefore, to understand the genetic mechanisms involved in WS, 15 functional candidate genes were chosen to be analyzed by quantitative PCR (qPCR) in breast muscle of normal and WS-affected chickens. To this, the pectoral major muscle (PMM) of 16 normal and 16 WS-affected broilers were collected at 42 days of age and submitted to qRT-PCR analysis. Out of the 15 genes studied, six were differentially expressed between groups. The *CA2*, *CSRP3*, and *PLIN1* were upregulated, while *CALM2*, *DNASE1L3*, and *MYLK2* genes were downregulated in the WS-affected when compared to the normal broilers. These findings highlight that the disruption on muscle and calcium signaling pathways can possibly be triggering WS in chickens. Improving our understanding on the genetic basis involved with this myopathy might contribute for reducing WS in poultry production.

## Introduction

The White Striping (WS) is one of the most prevalent myopathies occurring in modern commercial broilers nowadays, which is characterized macroscopically by parallel white fatty striations across the muscle fibers on the surface of breast fillets and thighs (Kuttappan et al., 2013). The most prominent histological features associated to this condition are myodegeneration with regeneration, perivascular necrosis, interstitial inflammation, lipidosis, and infiltration of connective tissue (Kuttappan et al., 2013; Sihvo et al., 2014; Russo et al., 2015; Vanhatalo et al., 2021). From a meat quality standpoint, the WS disorder represents a huge economic impact on the chicken industry, since it affects the most valuable part of the broiler carcass, increasing the downgrading percentages and condemnation of breast fillets. Moreover, the abnormal appearance of the meat negatively affects the consumer acceptance and increases concerns about animal welfare (Kuttappan et al., 2012; Petracci et al., 2015; Ayansola et al., 2021). In 2019, the world poultry meat production reached 120 million tons with an average consumption of 50.7 and 20.8 Kg per capita in the United States (USA) and European Union (EU), respectively (Avec, 2020). Breast muscle disorders in the USA poultry industry have been estimated to cause economic losses ranging from $200 million to more than $1 billion dollars per year (Kuttappan et al., 2016; Barbut, 2019).

It has been hypothesized that genetic selection of commercial broilers for fast growth increases the incidence of myopathies, and a number of factors have been associated with the onset and severity of WS, such as genotype (high breast yield), gender (males), fast growth rate, heavy weight of the *Pectoralis major*, and diets with high energy (Petracci et al., 2013; Mutryn et al., 2015; Trocino et al., 2015; Cruz et al., 2017; Griffin et al., 2018; Vanhatalo et al., 2021). Different causative mechanisms for WS occurrence have been suggested to date: hypoxia, oxidative stress, fiber-type switching, disruption in satellite cells proliferation and differentiation, metabolic disorders, and nutritional deficiencies, but no conclusive evidence was found regarding the etiology of this disorder (Russo et al., 2015; Daughtry et al., 2017; Boerboom et al., 2018; Marchesi et al., 2019; Soglia et al., 2019; Abasht et al., 2021; Lake et al., 2021). Swatland (1990) suggested that selection for rapid growth has created muscle that outgrow their life support systems and cause muscle damage. Therefore, the formation of large intercellular spaces leads to loss of muscle fiber fluids, compromising the muscular integrity of chickens. Thus, the selected modern hybrids have differences in muscle histology and metabolism, with higher density of fast twitch fiber, which is characterized by a higher diameter and a lower rate of protein degradation, compared to unselected broilers (Picard et al., 2010).

Genetic studies have shown heritability estimates ranging from 0.18 to 0.65 for WS (Bailey et al., 2015, 2020; Alnahhas et al., 2016; Lake et al., 2021) indicating an important genetic component influence on the occurrence of WS in broilers. Furthermore, positive genetic correlations between WS and body weight (0.23) and breast yield (0.31) traits, as well as with other myopathies, such as wooden breast (0.74) and deep pectoral myopathy (0.34) have also been found (Bailey et al., 2020). Recently, many genes have been associated with the occurrence of myopathies in chickens (Zambonelli et al., 2016; Beauclercq et al., 2017; Zampiga et al., 2018; Marchesi et al., 2019; Lake et al., 2021), and the identification of specific biomarkers can help to accurately assess the effect of genetics, environment, or management conditions in improving or aggravating the WS myopathy in broilers (Beauclercq et al., 2017). Some candidate genes involved with the development of WS in fast-growing broilers suggest that these genes play important role in molecular mechanisms, such as muscle differentiation, oxidative stress, calcium signaling, hypoxia, and muscle fiber type replacement (Mutryn et al., 2015; Marchesi et al., 2019). Despite the studies searching for the causes of the WS development, efforts are still needed to understand and, consequently, reduce this problem. In a recent review by Soglia et al. (2021), the importance of gene expression studies to unravel the molecular mechanisms underlying the findings obtained by different techniques and approaches to understand myopathic disorders in a global manner was highlighted. Thus, gene expression studies evaluating normal and WS-affected tissues would provide additional support to find the triggering pathways of this myopathy. Therefore, the aim of the present study was to evaluate the expression profile of 15 functional candidate genes by quantitative PCR (qPCR) in the breast muscle of normal and WS-affected chickens to provide more insights on the molecular events underlying the occurrence of moderate levels of WS.

## Materials and Methods

### Experimental Animals and Tissue Collection

A total of 168 male broilers from the commercial line Cobb500 were raised at the Embrapa Swine and Poultry National Research Center farm, located in Concórdia, Santa Catarina State, Brazil. Chicks were vaccinated in the hatchery against fowl pox and Marek disease. The management conditions followed the guidelines of the line, with water and feed provided *ad libitum*. At 42 days of age, all broilers were weighed and slaughtered by cervical dislocation following the procedures of the Ethics Committee for Animal Use (CEUA) from the Embrapa Swine and Poultry National Research Center, under protocol No. 012/12.

Immediately after slaughter, a subsequent necropsy was performed, and breasts of all animals were visually evaluated for the presence and severity of WS and classified based on the 3 degrees of lesions established by Kuttappan et al. (2013). A total of 16 broilers with normal breasts and 16 with moderate degree of WS (fillets with white striations generally less than 1 mm thick on the surface of the breast) were chosen for this experiment. Samples from the cranial region of the pectoral major muscle (PMM) were collected by removing approximately 1 g of the breast muscle of each animal, which was immediately frozen in liquid nitrogen and stored at −80°C for further molecular analysis.

### RNA Extraction and cDNA Synthesis

Frozen samples were ground in liquid nitrogen, and 100 mg of the PMM was submitted to RNA extraction using Trizol (Invitrogen, United States) following the manufacturer’s protocol. Briefly, 1 ml of Trizol was added to the PMM, homogenized at room temperature (RT) for 5 min, and then 200 μl of chloroform was added and the samples were shaken vigorously. The tubes were centrifuged at 10,000 × *g* at 4°C for 15 min, and the aqueous phase was transferred to a microtube containing 500 μl of 100% isopropyl alcohol. Another RT incubation for 10 min was performed, followed by a 12,000 × *g* centrifugation for 10 min. The RNA pellet was washed with 75% ethanol and centrifuged at 7,500 × *g* for 5 min at 4°C. Pellets were dried for 15 min at RT and resuspended in DEPC-treated water. The total RNA was quantified in BioDrop spectrophotometer (Biodrop, United Kingdom). Samples with 260:280 nm ratio higher than 1.8 were considered pure. The integrity of the RNAs were confirmed in 1.5% agarose gel after electrophorese for 90 min. The first strand cDNA was synthesized using 4 μg of total RNA and SuperScript III First-Strand Kit SuperMix Synthesis (Thermo Fischer Scientific, EUA), using oligo dT primer, following the manufacturer’s recommendations.

### Real Time RT-PCR

A total of 15 functional candidate genes (Table 1) previously reported as potentialy involved with WS development in chickens were chosen from the literature to be evaluated for gene expression analysis (Mutryn et al., 2015; Marchesi et al., 2019). These genes participate in several biological processes important for muscle development and homeostasis, such as regulation of actin cytoskeleton, focal adhesion, nitrogen metabolism, hypoxia, calcium signaling pathway, and insulin signaling pathway. The gene sequences were obtained in the Genbank^1^ and Ensembl^2^ databases. Primers for each gene (Table 1) were designed in exon-exon junctions to avoid DNA amplification using the Primer-Blast online tool (Ye et al., 2012), and primer’s quality was evaluated in the Netprimer online tool.^3^

**Table 1:** Primers for the 15 candidate genes and reference genes used for the quantitative PCR (qPCR) analysis in the breast muscle of normal and White Striping (WS)-affected broilers.

Gene	Ensembl ID	Primer (5'–3')	Size (bp)
*ACTG1* Actin, gamma 1	ENSGALG00000028749	F: 5'-CTCTGTTCCAACCCTCTTTCCT-3' R: 3'-GTGTTGGCGTACAGATCCTTC-5'	112
*CA2*	ENSGALG00000030781	F: 5'-GCTCTGAGCAGATGTGCAAAC-3' R: 3'-CTCATCGCTGAGGTTACTGGAAG-5'	144
Carbonic anhydrase 2			
CALM2^*^ Calmodulin 2	ENSGALG00000010023	F: 5´-CCACCATGGCTGATCAACTG-3' R: 5´-GCCATTGCCATCAGCGTCTA-3´	191
*PLIN1* Perilipin 1	ENSGALG00000023395	F: 5'-GGCTATGGAGACGGTGGATG-3' R: 5'-CTGGCTTGCTCTCCTCTTCC-3'	173
*CHST1* Carbohydrate sulfotransferase 1	ENSGALG00000008440	F: 5'-CGCCCCTCTTTCTCGTCTTC-3' R: 5'-CTCATCGCTGAGGTTACTGGAAG-3'	133
*CSRP3* Cysteine and glycine rich protein 3	ENSGALG00000004044	F: 5'-GCTCTGAGCAGATGTGCAAAC-3' R: 3'-GCTTGGAGAGACCCGATTCC-5'	202
*CTGF* Connective tissue growth factor	ENSGALG00000037402	F: 5'-TCACCAACGATAATGCTTTCTG-3' R: 3'-GAATGCACTTTTTGCCTTTCTT-5'	111
*DNASE1L3* Deoxyribonuclease I-like 3	ENSGALG00000005688	F: 5'-GAGTTTGCGTGGCTCATCG-3' R: 3'-CACGATCCTGTCATAGGGGC-5'	78
*HDAC1*	ENSGALG00000003297	F: 5'-GGGGCGGGTTGCGTT-3'	115
Histone deacetylase 1		R: 3'-ACATCACCGTCGTAGTAGTAGC-5'	
*HIF1A* Hypoxia inducible factor 1 alpha subunit	ENSGALG00000011870	F: 5'-CGTCACCGACAAGAAGAGGATT-3' R: 5'-GTCAGCCTCATAATGGATGCCT-3'	171
*MAPK13*	ENSGALG00000030966	F: 5'-TCTGCTCCGCCATAGACAAG-3' R: 5'-CAAGCAGCCCAATGACATTCTC-3'	150
Mitogen-activated protein kinase 13			
*MYLK2*	ENSGALG00000006273	F: 5'-ACCCTTTTGAGATATTGGACGA-3' R: 5'-TCCTTGGAGCTGAGGTTGTACT-3'	112
Myosin light chain kinase 2			
*RYR2*	ENSGALG00000010812	F: 5'-ATACAAGGGACCTGCTGGGT-3' R: 5'-GGGAGGCAAAACAATCTGGC-3'	92
Ryanodine receptor 2			
*SMAD3* SMAD family member 3	ENSGALG00000035701	F: 5'-CCCCATGTCATCTACTGCCG-3' R: 5'-GGTAACACTGGGGTCTCCAC-3'	158
*TNNC2*	ENSGALG00000006835	F: 5'-GTCAATGACGGACCAGCAG-3' R: 5'-CGTCCGCATCAAACATGTCA-3'	95
Troponin C2, fast skeletal type			
RPL5^**^ Ribosomal protein L5	ENSGALG00000005922	F: 5'-AATATAACGCCTGATGGGATGG-3' R:5'-CTTGACTTCTCTCTTGGGTTTCT-3’	99
*RPL30* Ribosomal protein 30	ENSGALG00000029897	F: 5'-ATGATTCGGCAAGGCAAAGC-3' R: 5'-GTCAGAGTCACCTGGGTCAA	273

F, forward (5'-3'); R, reverse (3'-5').

* Paludo et al. (2017).

** De Oliveira et al. (2017).

The qPCR reactions were amplified on an QuantStudio 6 (Applied Biosystems, Foster City, CA, United States), in a final volume of 15 μl containing 1x Maxima SYBR Green (Fermentas, United States), 0.13 μM of each primer, and 2 μl of 1:10 diluted cDNA. The reactions for all primers followed the cycling condition: 95°C for 10 min, 40 cycles of 15 s at 95 and 60°C. A melting curve stage of 70–95°C was added in all qPCR reactions to verify their specificity. Primer specificity was also confirmed by 1% agarose gel. The reactions were analyzed in duplicates, and negative controls were included to detect contamination. Primers efficiency were obtained by linear regression [efficiency = 10 ^(−1/slope)^], according to Livak and Schmittgen (2001), and primers with efficiency between 95 and 105% were considered for gene expression analysis.

### Differential Gene Expression Analysis

The average of cycle thresholds (Ct) values was collected, and the 2^-ΔΔCT^ was calculated for each sample to obtain the fold-change (Livak and Schmittgen, 2001). The *RPL30* (*Ribosomal protein 30*) and *RPL5* (*Ribosomal protein L5*) reference genes were used for normalization (Table 1). These reference genes were chosen based on their stability values previously evaluated in the PMM of normal and WS-affected broilers (Marciano et al., 2020). The comparison of the relative gene expression between normal and WS-affected groups was performed using Mann-Whitney test. Genes with values of *p* < 0.05 were considered differentially expressed (DE).

### Gene Interaction Analysis

The STRING online software^4^ was used to evaluate the interactions among the DE genes. This software predicts the evidence of gene interactions based on co-expression and co-localization. The gene network was constructed using both *Gallus gallus* and *Homo sapiens* databases, in order to improve the information about the interaction among genes.

## Results

From the total of 168 broilers evaluated in this experiment, 146 birds (87%) presented different degrees of macroscopic lesions associated with WS and 22 chickens (13%) showed normal breast muscle. The average body weight of the WS-affected and the normal groups were 2.89 kg and 2.85 kg, respectively.

**Figure 1:**
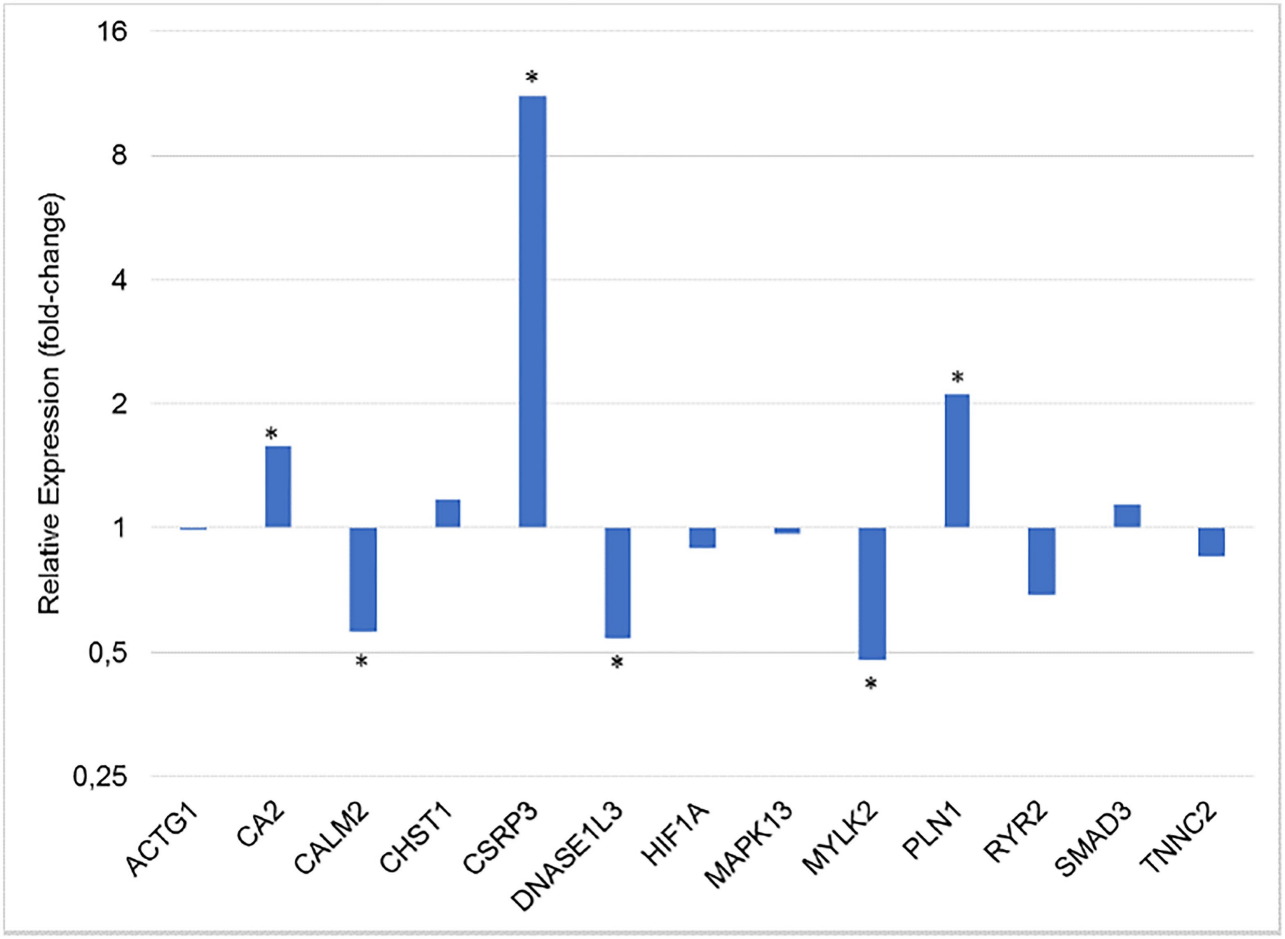
Gene expression ratio between normal and WS-affected broilers at 42 days of age, normalized for *RPL30* and *RPL5* reference genes. ^*^p < 0.05.

The relative expression of all 15 genes studied was obtained from the breast muscle tissue of normal and WS-affected 42 days-old broilers (Table 2). The *CSRP3* gene had the highest fold-change (14.56) and the others had a magnitude ranging from 1 to 2.11 (Table 2; Figure 1).

**Table 2:** Relative expression (fold-change) between normal and WS-affected broilers, respective *p* values, and expression levels calculated for each group.

Genes	Relative expression	*p* value	Fold-change by group (Mean ± SD)	
			Normal	WS-affected
*ACTG1*	−1	0.717	1.12 ± 0.49	1.12 ± 0.56
*CA2*	1.58^*^	0.006	1.07 ± 0.38	1.68 ± 0.75
*CALM2*	−1,79^*^	0.007	1.11 ± 0.55	0.62 ± 0.62
*CHST1*	1.18	0.945	1.57 ± 1.61	1.84 ± 2.92
*CSRP3*	14.56^*^	0.0001	0.84 ± 0.56	12.38 ± 12.21
*DNASE1L3*	−1.85^*^	0.005	1.11 ± 0.50	0.60 ± 0.34
*HDAC1*	−1.01	0.508	1.11 ± 0.48	1.02 ± 0.44
*HIF1A*	−1.12	0.151	1.12 ± 0.58	0.99 ± 0.85
*MAPK13*	−1.03	0.703	1.21 ± 0.83	1.17 ± 0.58
*MYLK2*	−0.8^*^	0.001	1.16 ± 0.91	0.36 ± 0.4
*PLIN1*	2.11^*^	0.009	1.47 ± 1.11	3.10 ± 1.97
*RYR2*	−1.45	0.773	1.57 ± 1.47	1.08 ± 0.67
*SMAD3*	1.14	0.816	1.37 ± 0.86	1.51 ± 1.26
*TNNC2*	−1.17	0.386	1.14 ± 0.71	0.97 ± 0.66

SD, standard deviation.

* *p* < 0.05.

Regarding the differentially expressed genes (*p* < 0.05), the *CALM2*, *DNASE1L3*, and *MYLK2* were downregulated in the WS-affected broilers when compared to the normal group (Figure 1). The expression levels of these three genes were, respectively, 1.79, 1.85, and 2.08 lower in WS-affected than in the normal broilers. The *CA2*, *CSRP3*, and *PLIN1* genes were 1.58, 14.56, and 2.11 more expressed in WS-affected broilers when compared to the normal group (Table 2; Figure 1). For the eight remaining evaluated genes, no differential expression was observed between groups (Figure 1).

**Figure 2:**
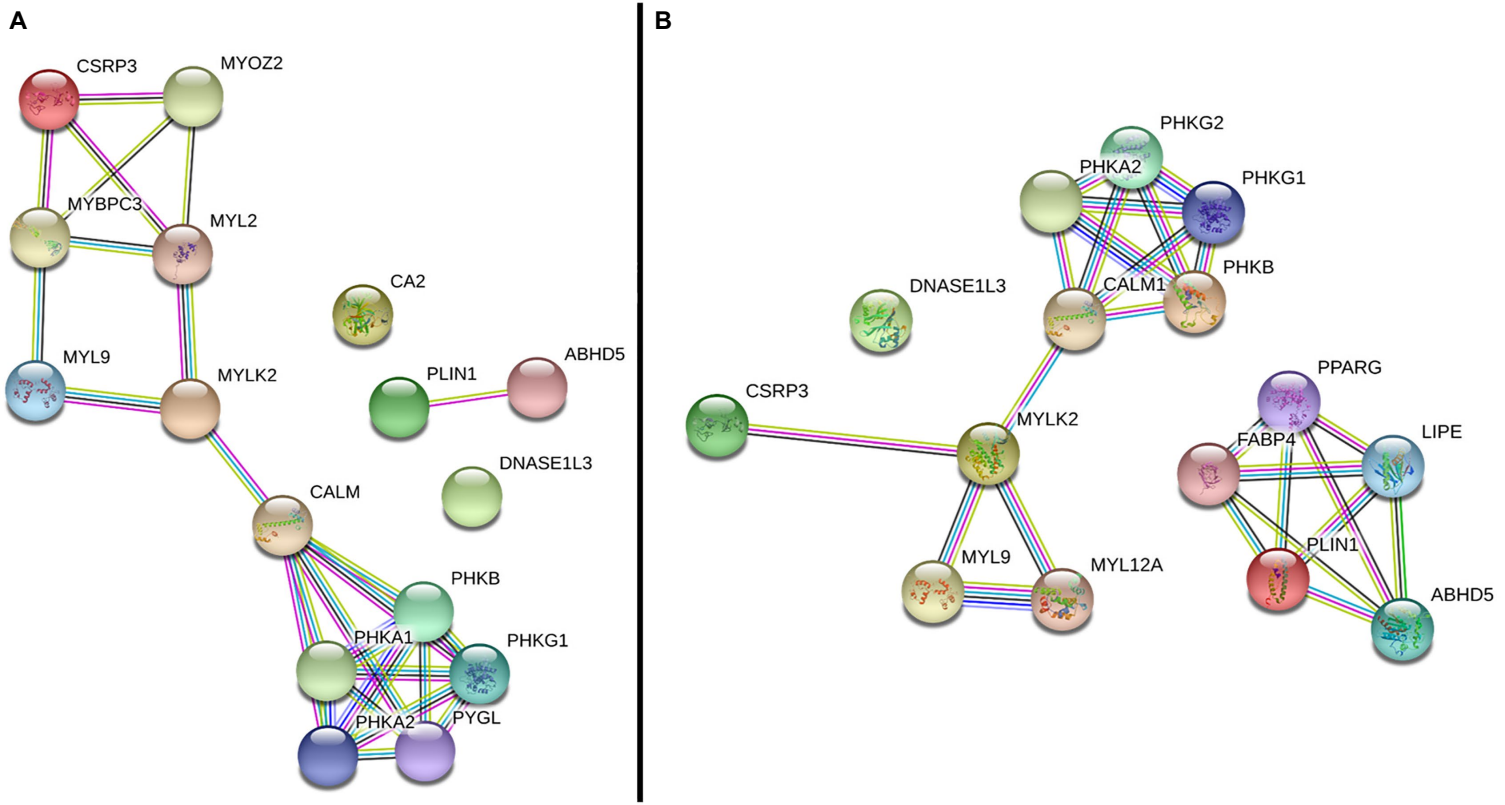
Gene network performed with differentially expressed (DE) genes between normal and WS-affected broilers, obtained with the String database using *Gallus gallus* **(A)** and *Homo sapiens* **(B)** protein information. Circles represent the genes. Lines represent the interaction among the DE and other related genes based on the prediction methods: known interaction in curated databases (light blue) and experimentally determined (magenta), predicted co-occurrence (blue), gene fusion (red) or neighborhood (green), homology (purple), text mining information (yellow), and co-expression (black).

In the gene network analysis performed with the six DE genes, the *CALM2* and *MYLK2* genes, which were downregulated in the WS-affected group (Table 2), were the main interactors of two gene clusters constructed from both chicken (Figure 2A) and human database (Figure 2B). One of the clusters was related to myogenesis and included the *CSRP3*, *MYOZ2*, *MYPBC3*, *MYL2*, and *MYL9* genes (Figure 2A), and the other was composed by phosphorylase kinase genic family (*PHKB*, *PHKA1*, *PHKA2*, *PHKG1*, and *PYGL*; Figure 2A), which play an important role in providing cell energy for muscle and liver tissue. The *CA2*, *DNASE1L3*, and *PLIN1* were not clustered with other genes according to the information available for *Gallus gallus* (Figure 2A). However, when the human database was used, the *PLIN1* gene was clustered with genes related to adipocyte differentiation, fatty acids, and glucose homeostasis (Figure 2B).

## Discussion

White Striping has been one of the main issues in poultry production in the last years. A number of studies have been conducted to understand the onset and development of WS, and other myopathies in chickens and some biological pathways have been associated to the occurrence of these conditions, such as extracellular calcium level, oxidative stress, localized hypoxia, possible fiber-type switching, and cellular repairing (Mutryn et al., 2015; Beauclercq et al., 2017; Kuttappan et al., 2017; Boerboom et al., 2018; Lake et al., 2019; Marchesi et al., 2019; Abasht et al., 2021; Bordini et al., 2021). In the present study, 15 functional candidate genes important for tissue development were investigated in normal and WS-affected broilers, and six of them (*CA2*, *CSRP3*, *PLIN1*, *CALM2*, *DNASE1L3*, and *MYLK2*) were differentially expressed.

The *CA2*, which was upregulated in WS-affected broilers in this study, is mainly involved in oxidative stress (Zimmerman et al., 2004). The *CA2* encodes zinc metalloenzymes and participates in a variety of biological processes, including calcification, acid-base balance, and bone resorption (Zimmerman et al., 2004). Genes of carbonic anhydrase (CA) family have regulatory and repair functions with the ability to act as an oxidant agent in both physiological and physiopathological conditions in skeletal muscle. This gene family is important to efficiently transport and eliminate carbon dioxide (CO^2^) from tissues (Chegwidden and Carter, 2000). Some genes of this family were reported as DE in muscle myopathies in broilers. Among them, *CA3* could be highlighted since it was approximately 25 times more expressed in chickens affected with Wooden Breast myopathy than in normal broilers (Mutryn et al., 2015). Nishita et al. (2012) also found that *CA3* was upregulated in the pectoral muscle of broilers with muscular dystrophy compared to normal chickens. The *CA4* gene is involved in the regulation of extracellular pH, reducing the muscular contraction and possibly increasing the intracellular acidosis (Peralta and Huidobro-Toro, 2016). The specific function of *CA2* associated to muscle development or myopathies, such as WS, has not yet been described, but it is also known that CAs are involved with lipid metabolism and obesity, and with the increase of mitochondrial oxidative stress (Supuran, 2012). Therefore, the upregulation of *CA2* in WS-affected broilers could be related to the adipose tissue deposition between muscle fibers. Nevertheless, the function of this gene should be further explored, since our results (Figure 1) differ from the expression pattern obtained by Marchesi et al. (2019), in which the *CA2* gene was downregulated in WS-affected broilers. Although both studies used 42 days-old broilers, we chose to analyze breast tissue with moderate levels of WS, whereas Marchesi et al. (2019) studied severe levels of the disorder. This discrepancy indicates that the expression of this gene could be modulated by the degree of WS lesions.

Oxidative stress is defined as the presence of metabolic and radical substances or so-called reactive (oxygen, nitrogen, or chlorine) species (Elnesr et al., 2019; Elwan et al., 2019). Regarding the oxidative stress in the skeletal muscle, the increase in reactive oxygen species (ROS) causes changes in the cell signaling pathways, affecting the release of calcium from the sarcoplasmic reticulum, resulting in damage to the contractile capacity of muscle cells (Allen et al., 2008). The increase in ROS is cytotoxic and can alter cell integrity, inducing severe stress and leading to the production of carbonic anhydrases signaling to the non-recoverable muscle damage (Zimmerman et al., 2004). In our study, the upregulation of the gene *CA2* in WS-affected broilers may have an oxidative action in the cell, which could lead to a hypoxic state in the tissue. This pattern has also been described in WB (Mutryn et al., 2015).

The *CSRP3* gene was upregulated in WS-affected broilers in our study. This gene encodes the LIM protein, which regulates important processes for the development and differentiation of satellite cells. In a very recent study of Wooden Breast in broilers, the upregulation of *CSRP3* reduced late proliferation of cultured satellite cells from birds before the WB appearance (Velleman et al., 2021). In the muscle, LIM interacts with various proteins, such as titin, participating on intracellular signaling cascades and in the maintenance of sarcomere integrity (Hershberger et al., 2008). Moreover, the LIM protein seems to be involved with stress response through compensatory signaling pathway. This protein appears to be essential to the structure and maintenance of the sarcomere (Shah et al., 2005) and its expression occurs mainly in slow skeletal muscle (Geier et al., 2008), helping the formation and growth of myotubes, which are key features for muscle repair (Schneider et al., 1999). Alterations in *CSRP3* expression profile changed the type of fibers from fast to slow in a study using rats as animal model (Geier et al., 2008). The upregulation of this gene has already been reported in PMM of chickens affected with myopathies, such as Wooden Breast (Mutryn et al., 2015) and WS (Marchesi et al., 2019).

According to Arber et al. (1994), the *CSRP3* promotes muscle differentiation, acting on regeneration, structural repair, and genetic regulation of the skeletal muscle. In WS-affected chickens, there is an increase in extracellular space because the muscle differentiation gradually leaves gaps between neighboring fibers and bundle fibers. In these gaps, the infiltration of mononuclear, adipose, and fibrous cells occurs and may be involved as a secondary response to myopathy (Barash et al., 2005). Offer and Cousins (1992) suggested that the upregulation of the *CSRP3* gene in chickens with myopathy is a repair mechanism of myofibers trying to regenerate the affected muscle. They also suggest that the positive regulation of *CSRP3* affects meat quality, since this gene is involved in the development of myofibers acting on the interchange of muscle fiber type. This gene has already been associated with different meat quality traits in bovine (Pierzchala et al., 2014) and porcine (Offer and Cousins, 1992; Xu et al., 2010), but no information is available in broilers. Moreover, *CSRP3* regulates the autophagy in muscle cells (He et al., 2014) and has already been associated with myopathies in humans (Rashid et al., 2015) and chickens (Picard et al., 2010), which make this gene a possible molecular marker for myopathies occurrence.

In the gene network (Figure 2), the *CSRP3* was grouped with several myogenic genes, including *MYLK2*, *MYL2*, and *MYOZ2*, which were DE in a previous chicken WS study (Marchesi et al., 2019). The *MYLK2*, a myosin light chain kinase gene, was downregulated in our study and it is essential for muscle contraction (Figure 1), composing the main myofibrillar proteins in muscle cells (Janin et al., 2018). This gene is a calcium/calmodulin dependent and it is responsible for light chain phosphorylation, facilitating its interaction with actin filaments and then inducing a contractile activity (Smith, 2010). *MYLK2* is expressed predominantly in fast skeletal muscle fibers, and studies with *MYLK2* knockout mice showed a decrease in the phosphorylation of the myosin regulatory light chain in the skeletal muscle (Park et al., 2011). Reduced levels of *MYLK2* in turkeys *pectoralis* muscle with PSE meat favored a low integrity of myofibrillar proteins reducing the skeletal muscle contraction (Zhi et al., 2005). In broilers, it has been observed that breast muscle with severe WS myopathy has low integrity of myofibrillar proteins, resulting in reduced water retention capacity compared to normal breasts (Malila et al., 2019) and, consequently, affecting meat quality (Alnahhas et al., 2016). The hypothesis is that the structural alteration of myosin facilitates water loss (Offer et al., 1989; Russo et al., 2015). Therefore, the presence of myopathies impairs the water holding capacity during the meat processing and storage (Barbut et al., 2005, 2008; Petracci et al., 2009). Furthermore, the reduced *MYLK2* mRNA levels in WS-affected chickens could lead to a decrease of the light chain phosphorylation, changing the myosin formation and muscle contraction. A similar pattern of *MYLK2* expression was observed in a study evaluating broilers affected with deep pectoral myopathies (Yalcin et al., 2018), showing a similar expression profile of this gene across different myopathies.

It is interesting to note that besides the *MYLK2*, the *CALM2* gene was also downregulated (−1.8) in WS-affected when compared to normal broilers (Table 2). *MYLK2* and *CALM2* were the two genes linking the main branches of the gene network, one composed by muscle development and the other by gluconeogenesis related genes, respectively (Figure 2). Calmodulin binds Ca^2+^ with high affinity and it is the most important Ca^2+^ signal transducer in the cells, regulating the activity of a plethora of proteins, such as kinases, transcription factors, and ion channels in most of the eukaryotes (Yalcin et al., 2018). The lack of Ca^2+^ transport to muscle fibers could increase the calcium concentration in the sarcoplasmic reticulum (Marchesi et al., 2019; Urrutia et al., 2019) affecting several biological processes, such as muscle contraction, oxidative stress, inflammation, and glycogen metabolism (Soike and Bergmann, 1998; Gronski et al., 2009; Choi et al., 2016; Yalcin et al., 2018). The disruption of calcium signaling pathway has been associated with different myopathies in humans (Sanmartín et al., 2017) and in chickens (Mutryn et al., 2015; Marchesi et al., 2019). In our study, low levels of *CALM2* could affect myogenesis, since the intracellular calcium concentration is essential for myosin light chain phosphorylation (Zhu et al., 2012). Moreover, reduced levels of *CALM2* could lead to insufficient intracellular calcium transportation and, consequently, to a defective muscle contraction ratio (Yalcin et al., 2018).

In the current study, the *CALM2* gene was also grouped with several genes from phosphorylase kinase family (PHKs; Figure 2). This family is responsible for the phosphorylation of certain muscle substrates, such as troponin I (Zhou et al., 2013) and it is involved in the glycogen metabolism/catabolism. The carbohydrate metabolism is an essential part of the skeletal muscle physiology. In humans, several metabolic myopathies have been associated with the impairment of carbohydrates and lipid metabolism (DiMauro and Spiegel, 2011). Some of them are characterized as disturbances in the glycogenolysis, affecting the glycogen metabolism (Zhou et al., 2013; Gaspar et al., 2019; Kishnani et al., 2019). Reduced levels of calmodulin could be involved with deficiency of PHKs, which could prevent the catalysis of glycogen in G-6-phosphate. Some *PHK* genes have already been associated with myopathies in chickens (Zambonelli et al., 2016; Marchesi et al., 2019), which reinforces the hypothesis that calcium signaling and carbohydrate metabolism genes are possibly involved with the onset of chicken myopathies.

In our study, the expression of the *DNASE1L3* gene was also reduced (Figure 1) in the WS-affected group. This gene is a member of the *DNASE1* family, which acts on the DNA catabolic processes, regulating inflammatory response, cytotoxicity, and hypoxia and presenting key functions related to tissue structure and development (Haller and Vissing, 2002; Napirei et al., 2005; Shi et al., 2017). The *DNASE1L3* is also known as DNASE gamma, being part of an endonuclease gene family that depends on Ca^+2^ and Mg^+2^ ions to be activated, usually participating in DNA cleavage and apoptosis (Shiokawa et al., 2002; Wu et al., 2018). It is known that apoptosis is an important process involved in skeletal muscle development in vertebrates, since it is necessary during the myogenic differentiation (Wu et al., 2018). The activation of *DNASE1L3* is considered to be responsible for DNA fragmentation needed to myoblasts differentiation (Wu et al., 2018). Therefore, the downregulation of this gene in the WS-affected broilers could prevent the occurrence of the correct myogenesis and consequently the cell hyperplasia. Another gene of this family, the *DNASE1L1*, was related to Pompe’s disease in humans, which is a glycogen storage disease characterized by muscle weakness (Perry and Rudnick, 2000). In chickens, there are few studies involving *DNASE1L3* gene function, especially considering its involvement with WS. Therefore, the association of this gene with WS development should be further investigated, since it is involved in important pathways associated to this condition. Furthermore, its calcium ion dependency could indicate some relationship between the downregulation of *CALM2* and *DNASE1L3*, narrowing the hypothesis that the dysregulation of calcium signaling is one of the most important pathways contributing to WS myopathy.

The *PLIN1* gene was twice upregulated in broilers affected with WS compared with the normal broilers (Figure 1) and, in the gene network analysis; this gene was not grouped with the main network in chickens neither in humans (Figure 2). The PLIN1 is one of the main proteins found in adipocytes, being required for the maintenance of the lipid metabolism, and normally inhibits the lipolysis of the cell (Haller and Vissing, 2002; Napirei et al., 2005; Kozusko et al., 2015). *PLIN1* has already been associated with intramuscular fat in pigs (Tansey et al., 2001) and lipodystrophy in humans (Kozusko et al., 2015). In chickens, it was previously described that retinoid X receptor α (*RXRα*) can promote adipogenesis by upregulating *PLIN1* expression (Sun et al., 2020). Furthermore, a RNA sequencing analysis was performed on pectoralis muscle samples of chickens with high and low triglyceride content and the *PLIN1* gene was differentially expressed between groups, suggesting that a high lipid deposition occurs in animals with high levels of triglycerides (Liu et al., 2019). Therefore, the upregulation of *PLIN1* in WS-affected broilers could explain the adipocyte differentiation and fat deposition between muscle fibers.

Finally, the other genes evaluated in this study were not differentially expressed between groups, although they participate in biological functions associated with myopathies, such as hypoxia, oxidative stress, immune response, and cellular repair. Nevertheless, previous studies have shown that some of those genes were related to chicken myopathies (Zambonelli et al., 2016; Marchesi et al., 2019). The discrepancy in these results could be due to several factors, such as high variation within the studied groups, the low magnitude of fold-changes, difference in the methodology used, the study of a single myopathy at a time and, more importantly, to differences in the severity of the myopathies, as shown by Malila et al. (2020). Furthermore, reporting expression profiles in different genetic groups, age and levels of white striping contribute to increasing the data on chicken myopathies and, consequently, mapping the main mechanisms involved in these conditions.

## Conclusion

The genes *CA2*, *CSRP3*, *MYLK2*, *CALM2*, *PLIN1*, and *DNASE1L3* were differentially expressed between normal broilers and those affected with moderate levels of WS at 42 days of age. These findings highlight that the disruption of muscle and calcium signaling pathways are involved in the development of WS in chickens. These results might be a reflection of the transition from moderate to severe levels of lesions or even the contrary, toward recovery of the muscle. Time course studies using broilers affected uniquely by white striping are needed to unravel the evolution of this disorder over time. Improving our understanding on the genetic basis involved with this myopathy may help finding alternatives to reduce White Striping in poultry production.

## Data Availability Statement

The original contributions presented in the study are included in the article/supplementary material; further inquiries can be directed to the corresponding author.

## Ethics Statement

The animal study was reviewed and approved by the Ethics Committee on Animal Utilization of the Embrapa Swine and Poultry National Research Center, under protocol number # 12/2012.

## Author Contributions

JO, ML, and AI conceived and designed the experiment. JO and ML were responsible for the data collection. AI, CM, JM, KC, and IS performed the laboratory experiment. AI, CM, KC, and IS performed the data analysis. AI, CM, JO, LF, and ML interpreted the results and wrote the manuscript. All authors reviewed, edited, and approved the final manuscript.

## Conflict of Interest

The authors declare that the research was conducted in the absence of any commercial or financial relationships that could be construed as a potential conflict of interest.

## Publisher’s Note

All claims expressed in this article are solely those of the authors and do not necessarily represent those of their affiliated organizations, or those of the publisher, the editors and the reviewers. Any product that may be evaluated in this article, or claim that may be made by its manufacturer, is not guaranteed or endorsed by the publisher.

## Footnotes

^1^https://www.ncbi.nlm.nih.gov/genbank/

^2^https://www.ensembl.org

^3^http://www.premierbiosoft.com/netprimer

^4^https://string-db.org/
